# Solvent exposure of Tyr10 as a probe of structural differences between monomeric and aggregated forms of the amyloid-β peptide

**DOI:** 10.1016/j.bbrc.2015.11.018

**Published:** 2015-12-25

**Authors:** Pablo Aran Terol, Janet R. Kumita, Sharon C. Hook, Christopher M. Dobson, Elin K. Esbjörner

**Affiliations:** a Department of Chemistry, University of Cambridge, Lensfield Road, Cambridge, CB2 1EW, UK; b Department of Biology and Biological Engineering, Division of Chemical Biology, Chalmers University of Technology, Kemivägen 10, 412 96 Gothenburg, Sweden

**Keywords:** Amyloid-β, Aβ oligomer, Amyloid fibril, tyrosine fluorescence, Acrylamide quenching

## Abstract

Aggregation of amyloid-β (Aβ) peptides is a characteristic pathological feature of Alzheimer's disease. We have exploited the relationship between solvent exposure and intrinsic fluorescence of a single tyrosine residue, Tyr_10_, in the Aβ sequence to probe structural features of the monomeric, oligomeric and fibrillar forms of the 42-residue Aβ_1-42_. By monitoring the quenching of Tyr_10_ fluorescence upon addition of water-soluble acrylamide, we show that in Aβ_1-42_ oligomers this residue is solvent-exposed to a similar extent to that found in the unfolded monomer. By contrast, Tyr_10_ is significantly shielded from acrylamide quenching in Aβ_1-42_ fibrils, consistent with its proximity to the fibrillar cross-β core. Furthermore, circular dichroism measurements reveal that Aβ_1-42_ oligomers have a considerably lower β-sheet content than the Aβ_1-42_ fibrils, indicative of a less ordered molecular arrangement in the former. Taken together these findings suggest significant differences in the structural assembly of oligomers and fibrils that are consistent with differences in their biological effects.

## Highlights

• Acrylamide quenching of Tyr_10_ provides insights into Aβ_1-42_ aggregate structure.
• Tyr_10_ is solvent-exposed in Aβ_1-42_ oligomers, but protected in Aβ_1-42_ fibrils.
• Aβ_1-42_ oligomers exhibit less β-sheet content than fibrils.
• Monomer arrangement in oligomers and fibrils may explain their different toxicities.

## 1 Introduction

Alzheimer's disease (AD) and a range of related disorders are associated with the self-assembly, aggregation, and fibril formation of disease-specific peptides and proteins [1]; in the case of AD such processes are involved with aggregation of the amyloid-β (Aβ) peptide [2], [3]. In its fibrillar form, this peptide is the main proteinaceous component of the extracellular plaque deposits that are characteristic of AD pathology [4], [5], but in the brain Aβ also exists in a variety of monomeric and oligomeric forms [6]. It has been reported that soluble Aβ concentrations correlate more closely with dementia than the amount of amyloid plaques [7], [8], and indeed soluble Aβ oligomers are now thought to be the main culprits in the pathogenesis of AD and related conditions [9], particularly since they have been associated with impaired cognitive function [10], [11], [12] and have been shown to induce cellular toxicity [13], [14], [15], [16], [17]. Unfortunately, the small size and low abundance of Aβ oligomers, in combination with their considerable heterogeneity and high sensitivity to environmental changes, has rendered them challenging to characterise. Although a detailed molecular model of a stabilised Aβ_1-42_ protofibril has been recently reported [18], little is known in detail how monomers arrange to build up the smaller, globular oligomers that are populated during Aβ aggregation reactions and which can form in the AD brain [19].

**Fig. 1:**
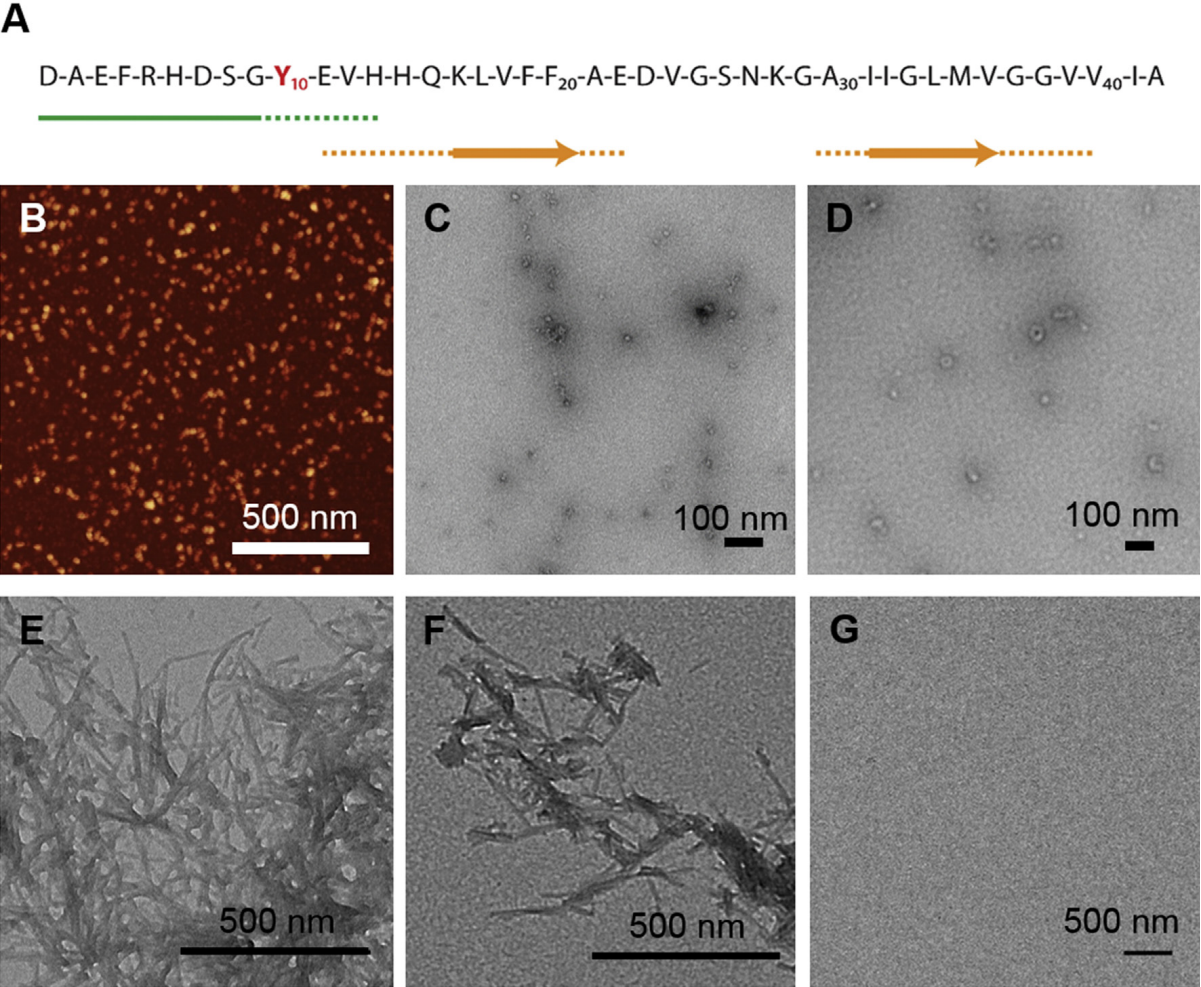
(A) Schematic representation of the Aβ_1-42_ peptide sequence. The arrows show the reported engagement of different parts of the sequence in the mature amyloid fibrils [24], [25], [26], [27], [28], with the unstructured N-terminus marked in green and the β-sheet regions participating in the core marked in orange. Tyr_10_ is highlighted in red. (B) Atomic force microscopy image of oAβ_1-42_. (C–D) TEM image of oAβ_1-42_ at higher magnification. (E) TEM image of fAβ_1-40_. (F) TEM image of fAβ_1-42_. (G) TEM image of mAβ_1-40_ showing the absence of aggregates in the monomeric preparation. (For interpretation of the references to colour in this figure legend, the reader is referred to the web version of this article.)

In the present paper we have used fluorescence and circular dichroism spectroscopies to examine structural differences, particularly related to the microenvironment around the N-terminal region, to compare soluble, globular Aβ_1-42_ oligomers, prepared *in vitro*, with monomers and fibrils formed by both Aβ_1-40_ and Aβ_1-42_. The oligomers were prepared using established methods for producing stable oligomers of a type that is often referred to as amyloid-derived diffusible ligands (ADDLs) [19]. These ADDLs have been reported to be neurotoxic [20] and it has been found that antibodies raised against *in vitro* prepared oligomers of this type also recognise oligomeric species that are elevated in AD brains [21], suggesting their resemblance to naturally occurring forms. Although it has been proposed that oligomers of this type could be of a fibrillar nature [22], [23], it is not known how monomers are present within these oligomer assemblies or to what extent the microscopic architecture resembles that found in mature fibrils. To address this issue, we have taken advantage of the fact that the Aβ peptide contains only a single intrinsically fluorescent residue, tyrosine at position 10 (Tyr_10_), which is located in between the β-core and the flexible N-terminus of fibrillar Aβ_1-40_ and Aβ_1-42_ [24], [25], [26], [27], [28] (Fig. 1A). This residue, therefore, has the potential to report on the participation of this region of the Aβ peptide in the different aggregated states. In this study, we have exploited the intrinsic fluorescent properties of Tyr_10_ and also its susceptibility to fluorescence quenching by water-soluble acrylamide to examine the characteristics of the monomeric, oligomeric and fibrillar forms of Aβ. Our results indicate that Aβ_1-42_ oligomers show the presence of a degree of β-sheet structure, but that they are distinctly less ordered than fibrils, and this is confirmed by a substantially higher degree of solvent exposure around Tyr_10_. Taken together these results highlight significant differences in the molecular architecture of the oligomeric versus the fibrillar forms of the Aβ peptides which may offer new insights into the differential biological activities.

## 2 Materials and methods

### 2.1 Materials

Synthetic Aβ_1-40_ and Aβ_1-42_ peptides were acquired as lyophilised powders from Anaspec EGT (Fremont, USA) and were prepared for use as described below. All other reagents were purchased from Sigma–Aldrich (Dorset, UK).

### 2.2 Sample preparation

The Aβ peptide powders were dissolved in ice-cold trifluoroacetic acid, sonicated (30 s, on ice), flash frozen and again lyophilised. The samples were redissolved in ice-cold hexafluoroisopropanol (1 mL) and the solutions were kept on ice (10 min) then divided into aliquots (50 μL) whilst working at 4 °C, and dried by rotary evaporation. The peptide concentration was determined by amino acid analysis and all experiments were performed in 50 mM sodium phosphate buffer (pH 7.4). The monomeric form of the Aβ peptides was obtained by dissolving a fresh peptide aliquot to a final concentration of 5 μM directly in buffer. The solution was analysed immediately to minimise the formation of aggregates. Fibrillar Aβ samples were prepared by incubation at room temperature for 48 h under shaking conditions (1400 rpm in a Titramax 100 shaker, Heidolph Instruments GmbH, Schwabach, Germany). Oligomers of Aβ_1-42_ were prepared by resuspending peptide aliquots in DMSO (2 μL), followed by dilution in ice-cold buffer (10 mM NaCl, 10 mM sodium phosphate, pH 7.4), to a final peptide concentration of 100 μM followed by incubation (overnight, 4 °C under quiescent conditions) [20]. The solutions were diluted in 50 mM sodium phosphate buffer (pH 7.4) (5 μM based on monomer concentration) prior to experimental analysis. The oligomer yield was determined by amino acid analysis following separation of the oligomers from residual monomer by ultracentrifugation (1 h, 90,000 g) using an Optima TLX ultracentrifuge (Beckman Coulter Inc, Brea, USA).

### 2.3 Fluorescence spectroscopy

Fluorescence spectra were recorded on a Cary Eclipse fluorimeter (Agilent Technologies, Stockport, UK) using a reduced pathlength quartz cuvette (4 mm excitation/10 mm emission). The excitation wavelength was 275 nm and emission spectra were recorded at 1 nm increments between 290 and 350 nm, with excitation and emission slit widths of 5 nm and 10 nm, respectively, and a scan rate of 60 nm/min. Samples were subjected to an acrylamide gradient following additions of a 0.2 M stock solution (10 μL aliquots), the fluorescence spectra being recorded immediately after the addition of the acrylamide aliquots. The recorded spectra were corrected for the background contributions and the fluorescence intensity in each case was taken as the sum of the intensities in a 6 nm range centred around the emission maxima, to increase signal-to-noise. Data were analysed using the Stern-Volmer equation [29].

(1) $$\frac{F_{0}}{F}=1+K_{SV}\left[Q\right]$$

where F_0_ and F are the fluorescence intensities in the absence and presence of quencher, [Q] is the concentration of quencher, and K_SV_ the Stern–Volmer constant. All experiments were performed in triplicate and are reported as mean ± SD. As acrylamide absorbs significantly at the excitation wavelength of tyrosine and therefore acts as an inner filter, corrections were made using separate experiments in which a non-quenching molecule (here DNA) was titrated into solutions of tyrosine at the same absorbance increments in order to determine correction factors for primary inner filter effects.

### 2.4 Circular dichroism (CD) spectroscopy

CD spectra were recorded on a JASCO J-810 spectropolarimeter (JASCO Inc. Tokyo, Japan) between 190 and 250 nm using a 1 mm quartz cuvette. 10 scans were recorded and averaged using a bandwidth of 2 nm and a scan speed of 50 nm/min. The peptide concentration was 30 μM and all spectra were corrected for background contributions by subtracting buffer blanks.

### 2.5 Transmission electron microscopy (TEM)

Aβ samples were adsorbed (2 min) onto carbon-coated copper grids (Taab Laboratories Equipment Ltd, Berks, UK). The grids were blotted, washed with milliQ water (2X) and negatively stained with 2% (w/v) uranyl acetate. Samples were imaged on a FEI Tecnai G2 transmission electron microscope (Eindhoven, Netherlands) and images were analysed using the SIS Megaview II Image Capture system (EMSIS GmbH, Muenster, Germany).

### 2.6 Atomic force microscopy (AFM)

AFM measurements were performed using a NanoWizard AFM system (JPK Instruments AG, Berlin, Germany). Samples were diluted using dH_2_O and deposited onto freshly cleaved mica surfaces and slowly dried before imaging. AFM imaging was carried out in the intermittent (air) contact mode using a silicon nitride cantilever (μmasch, NSC36/No Al, 65–130 kHz, 0.6–2 N/m). AFM images were analysed using Gwyddion software package (http://gwyddion.net/).

## 3 Results and discussion

The intrinsic fluorescence of Tyr_10_ in combination with circular dichroism spectroscopy has been used to examine five different preparations of Aβ_1-40_ and Aβ_1-42_; monomers (mAβ_1-40_ and mAβ_1-42_), fibrils (fAβ_1-40_ fAβ_1-42_) and Aβ_1-42_ oligomers (oAβ_1-42_) in order to assess the conformational differences between the various forms of Aβ_1-42_, and also to explore potential differences between Aβ_1-40_ and Aβ_1-42_ fibrils.

Prior to spectroscopic characterisation the morphology of the different Aβ species was examined (Fig. 1B–G). Analysis of the oligomeric oAβ_1-42_ samples by AFM (Fig. 1B) and TEM (Fig. 1C–D) showed that they contain a relatively homogeneous population of small aggregates with approximately spherical morphology; analysis of the TEM images indicates that their approximate diameters were 10–20 nm. The fibrillar fAβ_1-40_ and fAβ_1-42_ samples contained typical amyloid fibrils; importantly no such fibrillar structures were detected in the oligomeric samples used in this study and no discernible aggregates were observed in the monomeric samples (Fig. 1G,).

**Fig. 2:**
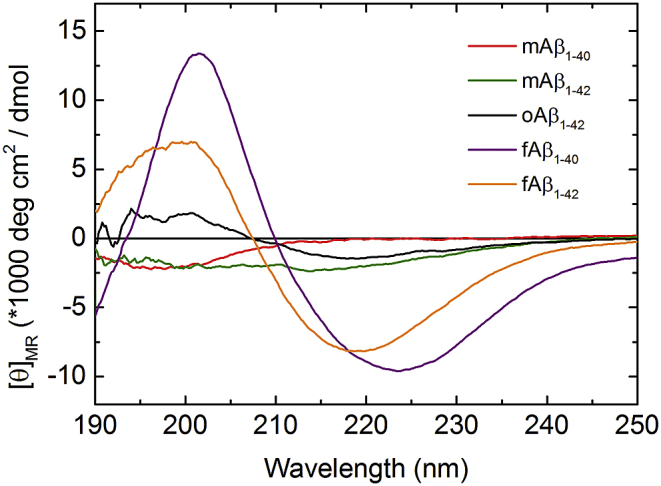
Circular dichroism spectra of different Aβ species; mAβ1-40 (red), mAβ_1-42_ (green), oAβ_1-42_ (black), fAβ_1-40_ (purple) and fAβ_1-42_ (orange). (For interpretation of the references to colour in this figure legend, the reader is referred to the web version of this article.)

Next, the secondary structure content in the different Aβ preparations was assessed (Fig. 2); the monomeric samples displayed CD spectra typical of random coils with negative peaks centred at 195 nm, consistent with their intrinsically disordered nature and confirming the absence of amyloid aggregates in the monomer preparations. The oligomeric and fibrillar samples, by contrast, exhibited characteristic β-sheet features with CD spectra displaying a negative peak at ∼218 nm and a positive peak at ∼196 nm. The CD signal from the oligomers, however, was considerably weaker than that of the fibrils indicating that they have significantly less β-sheet content.

**Fig. 3:**
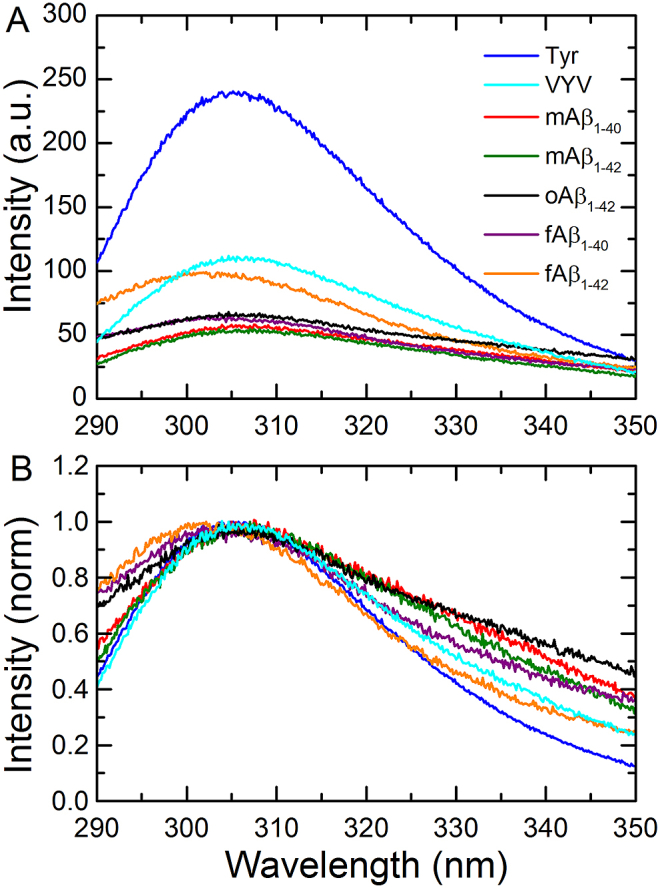
Intrinsic tyrosine fluorescence of different Aβ species. (A) Emission spectra of free tyrosine (blue), VYV (cyan), mAβ_1-40_ (red), mAβ_1-42_ (green), oAβ_1-42_ (black), fAβ_1-40_ (purple), fAβ_1-42_ (orange). (B) Normalised emission intensity, corresponding to the spectra shown in (A). (For interpretation of the references to colour in this figure legend, the reader is referred to the web version of this article.)

The fluorescence spectra of the various Aβ samples were recorded to monitor the intrinsic emission from Tyr_10_. Fig. 3A shows the spectra of the five different Aβ preparations along with corresponding spectra for free tyrosine and for a tyrosine-containing tri-peptide (VYV), the latter was used to determine the generic effects on tyrosine emission due to its incorporation into a polypeptide sequence. It was observed that the tyrosine emission intensity is decreased by as much as a factor of 2 upon incorporation into a peptide sequence, consistent with previous observations where the quenching of tyrosine in proteins has been attributed to photoinduced electron transfer to the peptide bond in presence of electron withdrawing groups [30]. All the Aβ conformers examined, except fAβ_1-42_, were observed to exhibit even lower intrinsic tyrosine fluorescence intensities than the VYV control peptide, indicating the existence of additional quenching interactions within the Aβ sequence, attributable to interactions with neighbouring side-chains. Spectral broadening of the tyrosine emission peak was observed in all the Aβ samples (Fig. 3B); the broadening on the blue edge of the emission peak is particularly apparent for fAβ_1-40_, fAβ_1-42_ and oAβ_1-42_ and can be explained by light scattering by the aggregates present in these solutions, whereas the red edge broadening, which is mainly apparent for the monomeric Aβ peptides, may be indicative of tyrosinate formation in the excited state [31], [32].

**Fig. 4:**
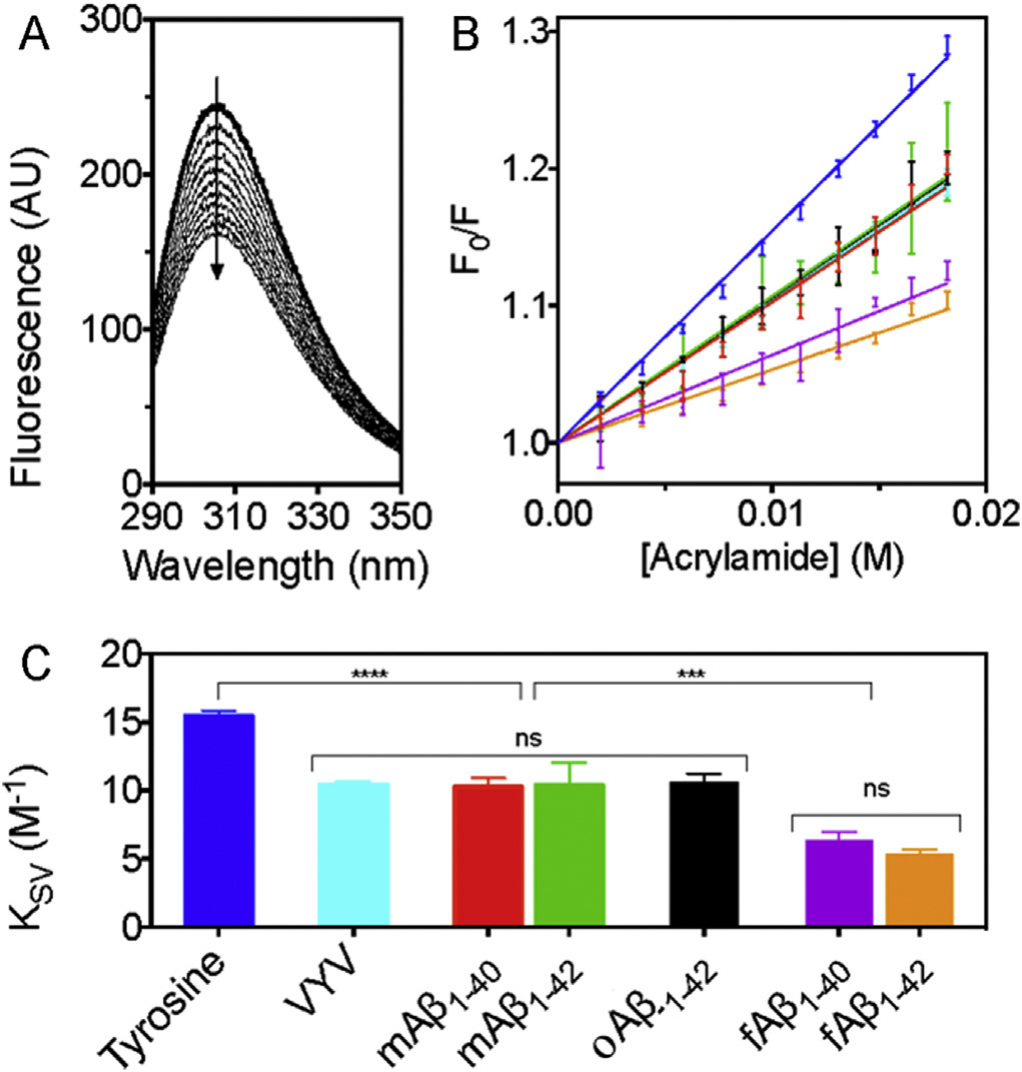
Acrylamide quenching of the intrinsic tyrosine fluorescence in the different species of Aβ. (A) Fluorescence emission spectra for free tyrosine (5 μM) titrated with acrylamide (added in 2 mM increments). The bold line represents the fluorescence of unquenched tyrosine and the arrow indicates the decrease in fluorescence with increasing concentration of the quencher. (B) Stern-Volmer plots of the acrylamide quenching of Tyr_10_ in the different Aβ species showing free tyrosine (blue), VYV (cyan), mAβ_1-40_ (red), mAβ_1-42_ (green), oAβ_1-42_ (black) fAβ_1-40_ (purple), fAβ_1-42_ (orange). The error bars represent the standard deviations (n = 3). (C) Stern–Volmer quenching constants (K_SV_) ±SD calculated from the linear-fit of the data in (B). The statistical significance of the differences between the various K_SV_ values was tested by one-way ANOVA with Tukey's post hoc test; *ns* denotes not significantly different (p > 0.05), *** (p < 0.001), **** (p < 0.0001). (For interpretation of the references to colour in this figure legend, the reader is referred to the web version of this article.)

Acrylamide quenching experiments were conducted to explore the extent to which tyrosine is exposed to solvent in its free form (Fig. 4A) compared to its exposure when it is incorporated as Tyr_10_ in the different monomeric and aggregated forms of Aβ. The resulting Stern-Volmer plots (Fig. 4B) were linear in all cases, an observation consistent with acrylamide being a predominately collisional quencher [33]. Fig. 4C summarises the calculated Stern–Volmer quenching constants (K_SV_) and shows that incorporation of tyrosine into a peptide sequence, even into the short VYV model peptide which displays a ∼30% reduction of the K_SV_ value, results in shielding from the polar but non-charged water soluble acrylamide quencher. This effect is likely to be due to steric hindrance imparted by the neighbouring residues, which limits the number of possible quenching interactions but does not reflect shielding caused by secondary structure constraints. No appreciable differences in the solvent exposure of tyrosine in the monomeric mAβ_1-40_ and mAβ_1-42_ forms compared to the VYV peptide were observed. This finding is consistent with other observations showing that the Aβ chain is highly unfolded in solution [34], [35], at least on timescales in the order of the excited state lifetime of tyrosine (≤3.4 ns [36]). Analysis of the Stern-Volmer plots showed, however, that Tyr_10_ in fAβ_1-40_ and fAβ_1-42_ is considerably less exposed to acrylamide quenching than in mAβ_1-40_ and mAβ_1-42_, suggesting that the incorporation of the Aβ molecules into a fibrillar structure protects this residue from solvent even though existing structural models [24], [25], [26], [27], [28] suggest that Tyr_10_ does not participate directly in the cross-β core (see summary in Fig. 1). This effect can, however, be explained by the close packing of monomer units in the fibril protofilaments [37], which sterically hinders the acrylamide-Tyr_10_ collisions, or it may also be a consequence of a fraction of the Aβ N-termini becoming buried within the mature fibril; indeed, limited proteolysis data for fAβ_1-40_ suggests that at least 20% of Aβ monomers have an N-terminal segment that is protected within the fibril structure [38]. The considerable similarity observed for fAβ_1-40_ and fAβ_1-42_ is interesting in relation to hydrogen/deuterium-exchange rates measured by NMR, which indicate significant differences between the two fibril types with respect to the amide solvent protection of the N-terminus, whereby the amide group of Tyr_10_ is more solvent accessible in fAβ_1-42_ than in fAβ_1-40_ [26].

Interestingly, the oligomeric oAβ_1-42_ samples exhibit K_SV_ values that are significantly higher than those observed for the fibrils (Fig. 4C). This observation suggests that Tyr_10_ is considerably less shielded in these globular oligomers than in Aβ fibrils and is consistent with our finding that oAβ_1-42_ species have a lower β-sheet content than fAβ_1-42_ (Fig. 2A). More surprisingly, we also find that the K_SV_ values of the oligomers are similar, within experimental error, to those of monomeric Aβ implying that Tyr_10_ is exposed to a similar extent as in a random coil monomer. To ensure that the high K_SV_ value truly reflects the structure of the oAβ_1-42_ species and is not attributable to sample heterogeneity, and as it has been suggested previously that oligomers of the type we examine here can exist in binary mixtures with monomers, a further control experiment was performed [39]. Quantitative amino acid analysis following ultracentrifugation to separate the oAβ_1-42_ species from residual monomers showed that, in our samples, nearly 90% of the Aβ monomers were incorporated into oligomers (Supplementary Information). This result is comparable to our previous report of the yield in fibril forming reactions with Aβ_1-40_ and Aβ_1-42_ [40]. Furthermore, these control experiments confirm that the difference in β-sheet content indicated in the oAβ_1-42_ and fAβ_1-42_ CD spectra, is indeed related to the oAβ_1-42_ species exhibiting some random coil nature, rather than due to the presence of significant quantities of unstructured monomers in the samples. Therefore, this study shows that the Aβ_1-42_ oligomers differ from fAβ_1-42_, not only in size and shape, but also in secondary structure content and internal architecture. Our finding that Tyr10 in the oligomers is significantly solvent exposed, and thus likely to be present on the surface of the oligomers is in agreement with a previous study of a disc shaped Aβ_1-42_ pentamer [41]. These observations are also consistent with the conclusion that the Aβ_1-42_ peptides in these oligomers do not adopt the same β-hairpin arrangement as that formed in the mature Aβ fibrils. This suggestion supports the idea that oligomers of this type may accumulate because their structural properties are such that they are not able to convert into fibrils [42], [43].

In conclusion, this study has set out to examine the fluorescent properties and the degree of solvent exposure of Tyr_10_ in Aβ to gain insights into structural similarities and dissimilarities between different Aβ species. We report significant differences between monomers and fibrils, suggesting that even though Tyr_10_ is not directly part of the cross-β core, the side-chain is, on average, well shielded within the fibrils. In addition, we have shown that Aβ_1-42_ oligomers, due to their lower β-sheet content and extensive exposure of Tyr_10_ to solvent, have significantly different structural properties from those of Aβ fibrils.
