# Supplementary material for: Solvent exposure of Tyr10 as a probe of structural differences between monomeric and aggregated forms of the amyloid-β peptide
Source: Biochem Biophys Res Commun. 2015 Dec 25;468(4):696–701. doi: 10.1016/j.bbrc.2015.11.018 (PMC4692451; doi:10.1016/j.bbrc.2015.11.018)
Supplement: Supplementary file 1 [file mmc1.pdf]

Suppoprting Information for

Solvent exposure of Tyr10 as a probe of structural differences between monomeric and aggregated forms of the amyloid-β peptide

Pablo Aran Terol[a], Janet R. Kumita[a], Sharon C. Hook[a,c], Christopher M. Dobson[a], Elin K. Esbjörner[a,b,*]

[a]Department of Chemistry, University of Cambridge, Lensfield Road, Cambridge, CB2 1EW, UK
[b]Department of Biology and Biological Engineering, Division of Chemical Biology, Chalmers University of Technology, Kemivägen 10, 412 96 Gothenburg, Sweden
[c]Present address: School of Biochemistry, University Walk, University of Bristol, Bristol, BS8 1TD, UK

*Corresponding author: E-mail: eline@chalmers.se, Phone: +46 (0) 31 772 5120

Quantification of monomer content in oAβ_{1-42} oligomers

In order to quantify the concentration of monomers that are incorporated into oligomers in the oAβ_{1-42} solutions we used ultracentrifugation to sediment the oAβ_{1-42} species and separate them from potential residual monomers. TEM analysis of the original oAβ_{1-42} solution, the supernatant and the pellet fractions confirmed the presence of oAβ_{1-42} in the initial solution and in the pellet, but not in the supernatant (Figure S1). The peptide content in the original solution, the pellet fraction and the supernatant was thereafter determined by quantitative amino acid analysis. The results showed that 89% of the peptide was in the pellet fraction corresponding to oligomers whereas, 11% remained soluble.

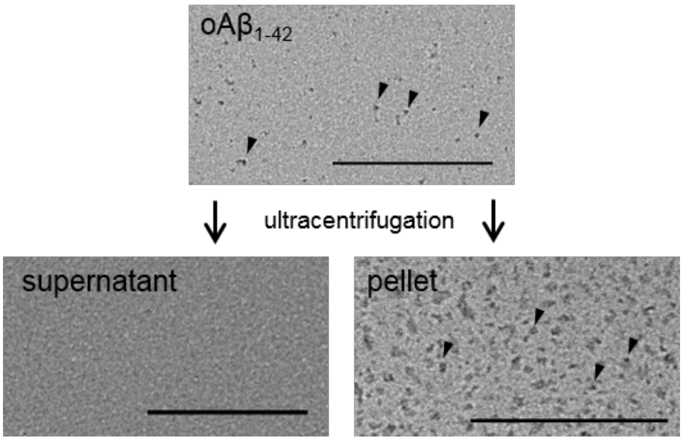

Figure S1. TEM images of oAβ_{1-42} before and after ultracentrifugation showing the presence of oligomers in the pellet fraction and absence of oligomers in the supernatant. Arrows indicate oAβ_{1-42}. The scale bars correspond to 500 nm.
